# Blast Injuries by an Improvised Explosive Device in Japan: A Case Report

**DOI:** 10.7759/cureus.32118

**Published:** 2022-12-01

**Authors:** Dai Kujirai, Ryo Fujii, Daiki Kaito, Rakuhei Nakama, Yoshimitsu Izawa

**Affiliations:** 1 Department of Surgery, Saiseikai Utsunomiya Hospital, Tochigi, JPN; 2 Department of Emergency Medicine, Ageo Central General Hospital, Saitama, JPN; 3 Department of Emergency and Critical Care Medicine, Keio University School of Medicine, Tokyo, JPN; 4 Department of Diagnostic Radiology, National Cancer Center Hospital East, Chiba, JPN; 5 Department of Emergency and Critical Care Medicine, Jichi Medical University, Tochigi, JPN

**Keywords:** disaster preparedness, mass casualty incident, triage, improvised explosive, blast injury

## Abstract

Blast injuries caused by an improvised explosive device (IED) are becoming more common in civilian settings. However, physicians may not be familiar with the treatment and management of blast-injured victims. To the best of our knowledge, this is the first case report of a blast injury caused by an IED in Japan.

A 64-year-old man was admitted to our hospital’s emergency department after sustaining a blast injury. His vital signs were stable, but he had multiple small wounds with embedded foreign bodies that were consistent with injuries sustained by IED victims. The patient was treated for his injuries and was moved to another hospital on day 37. Knowledge about blast injuries caused by IEDs and management strategies for mass casualties are both necessary.

## Introduction

Historically, blast injuries in the civilian population have been rare and were most often caused by an accidental explosion or fire. Recently, the number of blast-related injuries caused by incendiary devices is increasing, not only in the military but also in civilian settings [1]. Bombs called "improvised explosive devices" (IEDs) are increasingly deployed in terrorist attacks and are becoming a public concern [2-4]. The US Department of Defense defines IEDs as devices that are placed or fabricated in an improvised manner; that incorporate destructive, lethal, noxious, pyrotechnic, or incendiary chemicals; that are designed to destroy, disfigure, distract, or harass; and that often include materials from military sources [1,5]. IEDs can be used in roadside bombings, mines, explosively formed projectiles, and suicide bombings [6]. Given the widespread threat of terrorism, every physician involved with emergency care should be familiar with the treatment and management of blast-injured victims of terrorist bombings [7], as blast injury encompasses a unique combination of physical trauma. To the best of our knowledge, this is the first case report of a blast injury caused by an IED in Japan.

## Case presentation

A 64-year-old man with no known medical history was admitted to our hospital’s emergency department because of a blast injury he sustained from an IED while walking in a park. At the scene, four people were injured and triaged, one of them as black (Category 0) and the others as red (Category I). One of the patients, who had been triaged as "red," was placed on a backboard, immobilized, and transported by ambulance to our emergency department.

**Figure 1:**
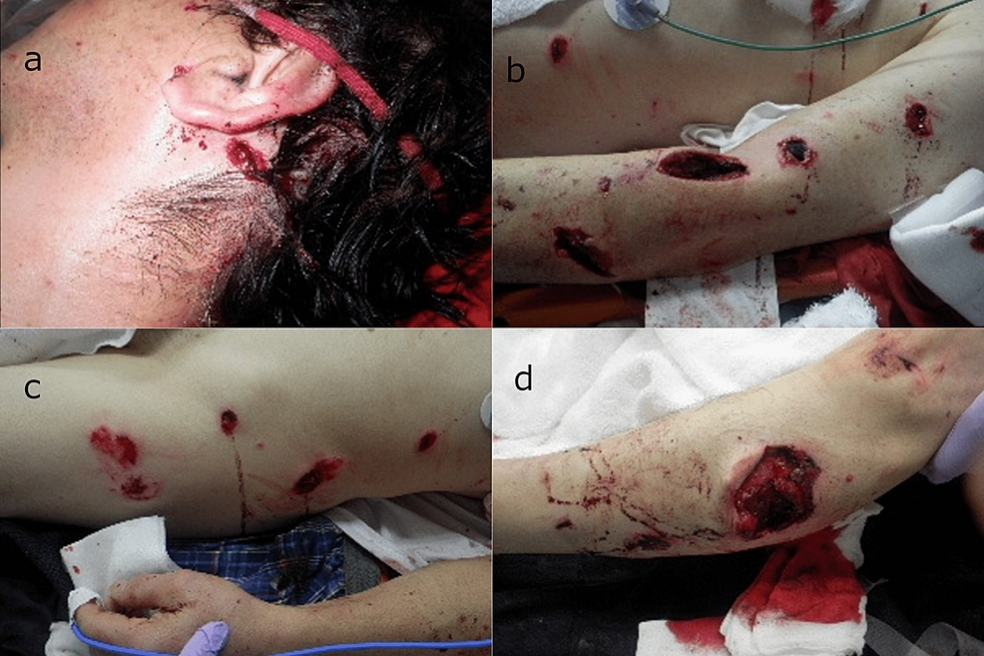
Multiple small wounds on the patient upon arrival. Multiple small wounds were present in the neck (a), left upper limb and left trunk (b and c), and left lower limb (d).

On presentation, the patient was alert and well-oriented, and his mental state registered 15 on the Glasgow Coma Scale. His blood pressure was 154/80 mmHg, his pulse was 78 beats per minute, his respiratory rate was 20 per minute, and his oxygen saturation was 97% (with an oxygen flow of 10 L per minute supplied by a nonrebreathing mask). He had multiple small wounds on his left side (Figure 1).

**Figure 2:**
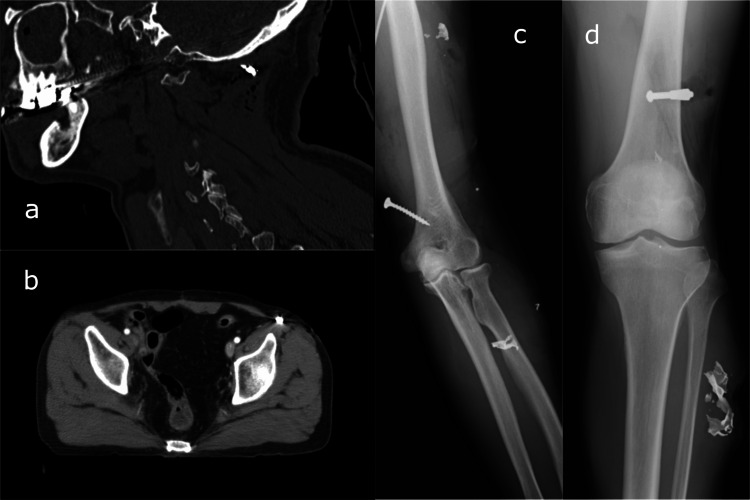
Computed tomography scan and radiograph of the patient. Foreign bodies such as nails and metal fragments were found in the neck (a), trunk (b), left upper limb (c), left lower limb (d).

He complained of hearing difficulty in his right ear and paresthesia in his left upper limb. As a part of the primary and secondary surveys, we performed a radiographic examination and computed tomography (CT) scanning. Multiple fragments of foreign bodies were found in his wounds, but none of them had penetrated the peritoneal, pleural, and cranial cavities (Figure 2).

A left tympanic membrane rupture was suspected but not visualized on otoscopic examination. As wound contamination was not so evident, we performed postexposure prophylaxis using tetanus toxoid. We determined the patient’s Injury Severity Score (ISS) to be 11 and his Revised Trauma Score (RTS) to be 7.841.

In the operating room, all wounds were well irrigated and debrided, and foreign bodies were removed as far as possible. The patient’s postoperative course was mostly uneventful. His paresthesia persisted, and a CT myelography on hospital day nine revealed a left brachial plexus injury. On hospital day 37, the patient was moved to another hospital for further rehabilitation.

## Discussion

Blast injury is currently recognized as having four different mechanisms of injury [1], although recently, a fifth mechanism has been proposed [8]. Primary blast injuries consist of the blast overpressure and transmitted force, which cause direct tissue damage. This force has a concentrated effect on the air-tissue interface and tends to be greater in closed spaces than in open spaces. Secondary blast injuries consist of debris displaced by blast overpressure and cause a combination of penetrating and blunt trauma. Tertiary blast injuries consist of the displacement of a casualty by the force of peak overpressure and blast winds, which cause closed head injuries, blunt abdominal trauma, tissue contusions, or fractures. Quarternary blast injuries consist of miscellaneous injuries not classified in the previous three mechanisms. They include burns, asphyxia from toxic substances, and psychological trauma. Quinary blast injuries are characterized by a hyperinflammatory state caused by blast injuries that is unrelated to the complexity or severity of the trauma. Such knowledge of blast injuries is essential in the management of each patient. Most of our case's injuries derived from secondary blast injuries, but an immediate head-to-toe assessment is necessary to rule out other injuries. One major reflecting point was that initial triage and decontamination of the patient should be performed outside the emergency department.

Initial assessment of blast-injured patients may be possible with the SALT (sort-assess-lifesaving interventions-treatment/transport) triage system [9], and portable otoscopes and pulse oximetry equipment may be useful for possible blast-injured patients in further assessment [10]. We should also keep in mind that the treatment of penetrating trauma from IEDs does not always require immediate surgical excision of shrapnel, although delaying treatment for several years has some risk of later complications [11]. Removal of shrapnel is recommended only when it involves intra-articular, neurovascular, or weight-bearing areas or the toxic effects of metals such as lead are expected. We removed the shrapnel as we could not exclude the toxic effects of the unknown metals.

Previously, blast injuries caused by explosive devices were seen mostly on the battlefield, but they have increasingly been seen in civilian settings. IEDs have been used in terrorist bombings around the world and have caused considerable damage. It may be necessary to manage many injured patients simultaneously in a mass casualty incident. In such a situation, details about the explosive device, geography, victim, and status of other casualties are very important information for patient risk stratification [12]. We had to consider chemical, biological, radiological, nuclear, or explosive (CBRNE) agents and assess the need for decontamination before the patient's arrival. Early recognition and appropriate triage for CRBNE casualties are needed to minimize secondary contamination [13]. Furthermore, blast exposure has recently been identified as an independent factor influencing psychiatric symptoms other than post-traumatic stress disorder and mild traumatic brain injury [14]. In this respect, the long-term impact of a blast injury cannot be underestimated.

Our case was very similar to the cases of many of the victims of the Boston Marathon bombing in 2013 [15], the London attacks in 2005 [16], and the Manchester Arena bombing in 2017 [17]. In Boston, 118 patients were treated at the nine closest hospitals, with a total of 264 victims treated in 27 hospitals. In London, more than 700 people were injured, and more than 200 patients were transferred to the closest hospital. In Manchester, 153 people were injured, and most of them were transferred to 11 hospitals. We should keep in mind that such casualties typically arrive at hospitals within several hours. In this case, we rapidly shared information with other departments within the hospital and prepared for further patient arrivals. Four people were injured in this incident, but no other people were transported to our hospital after this patient’s admission. In this case, we took care to confirm that no other casualties from the blast incident had been admitted before proceeding to surgery.

## Conclusions

Blast injuries caused by IEDs may occur suddenly in civilian settings and could constitute a mass casualty incident. A management strategy for mass casualties is therefore essential for each healthcare system and should be determined and established as soon as possible, wherever none is currently in place. In addition, we must always consider the possibility of CBRNE incidents in such a situation.
